# Chronic Cholecystitis of Dogs: Clinicopathologic Features and Relationship with Liver

**DOI:** 10.3390/ani11113324

**Published:** 2021-11-21

**Authors:** Ikki Mitsui, Shigeaki Ohtsuki, Kazuyuki Uchida

**Affiliations:** 1 Laboratory of Veterinary Pathology, Faculty of Veterinary Medicine, Okayama University of Science, Imabari 794-8555, Japan; 2 Japan Institute of Statistical Technology, Adachi-ku, Tokyo 123-0843, Japan; s.ohtsuki@jiost.com; 3 Laboratory of Veterinary Pathology, Graduate School of Agriculture and Life Sciences, The University of Tokyo, Bunkyo-ku, Tokyo 113-8657, Japan; auchidak@g.ecc.u-tokyo.ac.jp

**Keywords:** cholecystitis, dog, gallbladder, histochemistry, histopathology, immunohistochemistry, liver, mucocele, pathology, statistics, ultrasonography

## Abstract

**Simple Summary:**

This study on the gallbladders and livers of 219 client-owned dogs provides a benchmark for future studies on chronic canine cholecystitis. The statistical evaluation of clinical data; histopathology; histochemistry; and immunohistochemistry in this report provides insight into the etiology of chronic cholecystitis in dogs

**Abstract:**

(1) Background: Chronic cholecystitis of dogs has not been vigorously investigated histopathologically. In addition, the relationship between gallbladder and liver diseases is not known. (2) Methods: We aimed to provide a hallmark for canine chronic cholecystitis using clinical data, histopathology, histochemistry, immunohistochemistry, and statistical analysis. (3) Results: Our investigation of 219 ultrasonographically abnormal surgically resected canine gallbladders revealed 189 cases (86.3%) of mucosal lymphoplasmacytic infiltration (chronic cholecystitis). Sludge, a gravity-dependent or nondependent fine granular hyperechoic material, was more prevalent (105/219, 47.9%) than mucocele (51/219, 23.2%) in this cohort. Mucosal lymphoid follicles were detected in 68/219 cases (31%), suggesting the influence of long-standing antigenic stimulation. Bacteria were histochemically detected in 41/60 (68.3%) of heavily inflamed gallbladders, 18/129 (14%) of lightly inflamed, and 3/18 (16.7%) of uninflamed gallbladders, suggesting a possible relationship between bacteria and chronic cholecystitis. Simultaneous liver biopsies revealed mild or no inflammation, changes consistent with primary portal vein hypoplasia, and mild hepatocellular degeneration. (4) Conclusions: Based on the results of our statistical analysis, we conclude that canine chronic cholecystitis is a long-standing inflammatory process of unknown (but possibly bacterial) etiology and that liver pathology is unlikely the cause of chronic cholecystitis in dogs.

## 1. Introduction

Major pathologies of canine gallbladders include inflammation (cholecystitis), bacterial infection, neoplasia, gallstone formation (cholelithiasis), rupture, and a massive accumulation of gelatinous mucus (mucocele) [1,2,3,4,5,6,7,8,9,10,11,12,13,14,15,16,17,18]. As ultrasonographic evaluation of this organ becomes common in veterinary practice, it is necessary to determine the relationship between abnormal imaging findings and histological changes in order to clarify the underlying pathogenesis and to predict disease outcomes [19]. The need for such knowledge is reflected in the increasing number of studies and publications related to sludge and mucocele in canine gallbladders [4,6,12,16,17,19,20,21,22,23,24,25]. Previously, veterinary practitioners regarded ultrasonographic findings of gallbladder sludge, gravity-dependent or nondependent fine granular hyperechoic content, as inconsequential incidental findings [4,20]. Recently, cholecystectomy of ultrasonographically abnormal gallbladders in asymptomatic canines has been given careful consideration [25] due to the fact that mobile sludge or mobile precipitate has been observed in eventually ruptured canine gallbladders [6]. Surgical intervention of such sludge or precipitate-containing gallbladders minimizes the risk of rupture resulting in perioperative death associated with bile peritonitis [26]. To provide more scientific knowledge to develop better treatment options for canine gallbladder diseases, especially those containing sludge, a detailed histomorphological evaluation of the diseased tissues is indispensable to better understand the underlying pathogenesis.

Cholecystitis of dogs may manifest itself in both acute and chronic forms. Acute canine cholecystitis has often been related to bacterial infection [18,27]. Ascending infection from the duodenum through the common bile duct and/or hematogenous infection via enterohepatic circulation are two major routes of infection with a paucity of histopathological evidence [27]. Cultures of canine gallbladder contents have identified various enteric bacteria such as *Escherichia coli*, *Enterococcus* sp, *Bacteroides* sp, and *Clostridium* sp [6,12,18,19,28,29]. Compared to the depth of understanding of acute cholecystitis, detailed investigation on canine chronic cholecystitis has been sparse [17,19]. A deeper understanding of the pathology of canine chronic cholecystitis is essential not only to establish an evidence-based clinical approach to this disease in veterinary medicine but also to elaborate diagnostic criteria for future communication between veterinary and medical experts.

We herein retrospectively investigated 219 sets of the gallbladder and liver tissue of client-owned dogs in Japan. All subjects had a variety of ultrasonographic abnormalities in the gallbladder. Our aims were (1) to closely describe histologic characteristics of canine chronic cholecystitis; (2) to compare findings of the gallbladders and livers to examine possible relationships between them; (3) to statistically evaluate the relationship between histologic changes and clinical factors to provide veterinarians with clinically useful information. 

## 2. Materials and Methods

### 2.1. Animals

We investigated surgically resected gallbladders from client-owned dogs in which abnormalities were detected by the attending veterinarians during ultrasonographic examination. Ultrasonographic diagnosis of mucocele was made using published criteria [1,30]. Sludge was diagnosed if gravity-dependent or -independent hyperechoic particulate material without acoustic shadowing was noticed. Liver biopsies were simultaneously obtained from the same dogs. Verbal or written consent was obtained from the dog owners before each surgical procedure by the attending veterinarians in all cases. The samples were submitted to No Boundaries Animal Pathology, LLC (formerly affiliated with Ikki Mitsui, Tokyo, Japan) for histopathological examination. Inclusion criteria of cases for our study were (1) paraffin blocks of both gallbladders and livers were available for histochemistry and/or immunohistochemistry; (2) samples had been obtained by July 2019 (starting the month of this study) back to April 2014 (starting month of operation of this laboratory). As a result, 219 cases fulfilled the criteria and were examined. Information on the subjects is summarized in Supplementary Table S1. Ultrasonographic descriptions of the gallbladder contents were given by the submitting veterinarians. The liver biopsy methods consisted of spooning (*n* = 98), wedge (*n* = 91), and en bloc (*n* = 30). Signalment and clinical information on submission sheets and intraoperative photographs and ultrasonographic images of gallbladder and/or liver were screened. All dogs were alive when the samples were surgically obtained. Of note, we did not examine tissues of autopsied dogs because bile imbibition tends to hamper a detailed histological examination, which is critical for our study. Since our study did not use any live animals, approval by the Institutional Animal Care and Use Committee (IACUC) was waived.

### 2.2. Histopathology

All samples were fixed in 10% neutral buffered formalin immediately after cholecystectomy and liver biopsy. Samples were embedded in paraffin wax after 1 to 5 days of formalin fixation. Four-micrometer-thick sections (2 to 5 sections from each gallbladder and 1 to 3 sections—1 from each accessible lobe—of the liver) were stained with hematoxylin and eosin (HE) for histopathological examination by the American/Japanese College of Veterinary Pathologists board-certified veterinary anatomic pathologist (Ikki Mitsui) according to the criteria presented in Supplementary Table S1. Gallbladder mucosal inflammation was histologically graded based on the number of lymphocytes and/or plasma cells in the lamina propria of the most crowded high-power field (0.237 mm^2^) in each specimen (evaluation was performed in multiple, up to 10 areas in a given specimen): grade 2 (G2)—equal to or more than 30 lymphocytes and/or plasma cells; grade 1 (G1)—lymphocytes and/or plasma cells are less than 30 but equal to or more than 1; grade 0 (G0)—no inflammatory cells are present. Mucocele was characterized by amorphous, amphophilic, thick gelatinous material that occupied the lumen and adhered to the hyperplastic mucosa. Hepatic lobular diameter (HLD; average of 3 measurements) and gallbladder wall total thickness (GWTT; measurement of 1 representative area) were measured on whole-slide images, prepared by a virtual slide scanner (NanoZoomer S210, Hamamatsu Photonics K.K., Shizuoka, Japan). The details of HLD measurement are described elsewhere [31]. GWTT was measured as the distance between mucosal and serosal surface levels in selected areas, avoiding extreme thickness or thinness in each specimen. Primary portal vein hypoplasia (PPVH) of the liver was diagnosed if all of the following features were present in a given liver specimen: (1) hypoplastic interlobular vein; (2) decreased HLD (smaller than 700 µm); (3) proliferation of interlobular arterioles/bile ductules. Liver inflammation was diagnosed if any one or a combination of the following was present, regardless of their severity: cholangitis, infiltration of inflammatory cells in the cholangiolar epithelium; pericholangitis, infiltration of inflammatory cells in the Glisson’s capsule (portal tracts) without breaching the limiting plate; hepatitis, invasion of inflammatory cells from portal tract into the hepatic parenchyma beyond the limiting plate. 

### 2.3. Histochemistry

Four-micrometer-thick sections of the gallbladders of 219 dogs were stained by Giemsa and Warthin–Starry methods following the established protocols. Presence and morphology of bacteria were recorded. 

### 2.4. Immunohistochemistry

Four-micrometer-thick sections of twelve G2 gallbladder samples (cases 19, 24, 35, 51, 52, 82, 99, 106, 108, 136, 146, and 172) were mounted and dried on Crest slide glasses (Matsunami Glass Ind., Ltd., Osaka, Japan) for immunohistochemistry (IHC). Primary antibodies used for IHC, their host, type, dilution, source, and catalogue number, are shown in Table 1. Slides were deparaffinized in xylene and rehydrated in graded alcohol solutions and water. Endogenous peroxidase was inhibited by immersion in 3% H_2_O_2_ in methanol for 20 min. Antigen retrieval was conducted by heating slides in a pressure cooker at 121 °C for 10 min in pH 6.0 citrate buffer (CD3, CD20, and MUM1) or in pH 9.0 EDTA buffer (Granzyme B). Nonspecific immunoreaction was blocked by incubating slides with 5% skim milk in phosphate-buffered saline for 20 min at room temperature. Reaction with primary antibodies was conducted at 4 °C overnight. Reaction with a secondary antibody, horseradish–peroxidase polymer-conjugated anti-rabbit and anti-mouse IgG (Histofine Simple Stain MAX PO MULTI, Nichirei Biosciences Inc., Tokyo, Japan), was conducted for 60 min at room temperature. Immunoreaction was visualized by applying diaminobenzidine solution (ImmPACT DAB Substrate, Vector Laboratories, Burlingame, CA, USA) and slides were briefly counterstained with hematoxylin. For control specimens, the following were used: tissue sections of normal canine lymph node (for CD3 and CD20), a confirmed case of canine cutaneous plasmacytoma (for MUM1), and a confirmed case of canine small intestinal LGL lymphoma (for Granzyme B), and all were simultaneously stained. IHC using these control specimens validated the above-mentioned procedures. 

### 2.5. Statistical Analysis 

Signalment, ultrasonographic, gross, and histologic findings were statistically analyzed using a commercially available software (SPSS Statistics 25, IBM Corporation, Armonk, NY, USA). Parameters included age, breed, sex, presence of sludge, mucocele, gallbladder mucosal inflammation (G0, G1, or G2), GWTT, bacteria, lymphoid follicles, edema, smooth muscle thickening, liver inflammation, HLD, and PPVH. Two questions were tested in this study: (1) how are the three grades of gallbladder mucosal inflammation, i.e., G0, G1, and G2, related to the above parameters? (2) With which parameters are mucocele and sludge related and how are they related? For question 1, in which three groups (G0, G1, and G2) were tested, continuous variables (HLD, GWTT, and age) showing normality and homoscedasticity were analyzed by a one-way analysis of variance (one-way ANOVA) and a round-robin, two-group comparison taking multiplicity into consideration. For continuous variables lacking normality, a nonparametric Kruskal–Wallis test was applied. Categorial data in assumption 1 were tested by the chi-squared test. For question 2, since mucocele and sludge are binary data (i.e., present or not), continuous variables were tested by t-test or Mann–Whitney’s U test while categorial data were tested by Fisher’s exact test or chi-squared test. For each analysis, the statistical significance level of 0.05 was used. As an exception, the significance level of a two-group comparison in multiple comparisons using the Bonferroni method was 0.017 (0.05/3).

## 3. Results

Please also refer to Supplementary Table S1.

### 3.1. Signalment (Age, Breed, Sex) and Chief Complaint

The average age was 10 y 7 months. The median age was 11 y, while the range was from 1 y 9 months to 16 y 11 months. Toy or miniature breeds were overrepresented in this study. In descending order, this cohort included Miniature Dachshund (*n* = 43), Toy Poodle (*n* = 41), Chihuahua (*n* = 27), mixed breed (*n* = 15), Miniature Schnauzer (*n* = 11), Shiba Inu (*n* = 11), Papillon (*n* = 9), Pomeranian (*n* = 9), Yorkshire Terrier (*n* = 8), Shetland Sheepdog (*n* = 6), Shih Tzu (*n* = 6), French Bulldog (*n* = 6), Maltese (*n* = 5), American Cocker Spaniel (*n* = 4), Pug (*n* = 4), Pembroke Welsh Corgi (*n* = 3), Jack Russel Terrier (*n* = 3), Beagle (*n* = 2), and one of each of Standard Dachshund, German Shepherd Dog, Golden Retriever, Labrador Retriever, Norfolk Terrier, and West Highland White Terrier. There were 114 spayed females (52.1%), 78 castrated males (35.6%), 18 intact males (8.2%), and 9 intact females (4.1%) in this cohort. Frequent clinical abnormalities included decreased appetite (*n* = 56), vomiting (*n* = 53), decreased alertness (*n* = 28), icterus (*n* = 21), diarrhea (*n* = 13), dental tartar (*n* = 5), pyrexia (*n* = 3), weight loss (*n* = 3), and hematuria (*n* = 2). Rare abnormalities included the following (one incidence for each): collapse, panting, harsh breathing, urinary incontinence, abdominal pain, abdominal swelling, polyuria, polydipsia, hematemesis, skin lesion, abnormal estrus cycle, cognitive disorder, and vulvar discharge. Ninety-one dogs did not show any clinical abnormality (41.5%). Clinical information of 25 dogs was not provided by the submitters.

### 3.2. Ultrasonographic/Gross Abnormalities of the Gallbladder 

**Figure 1:**
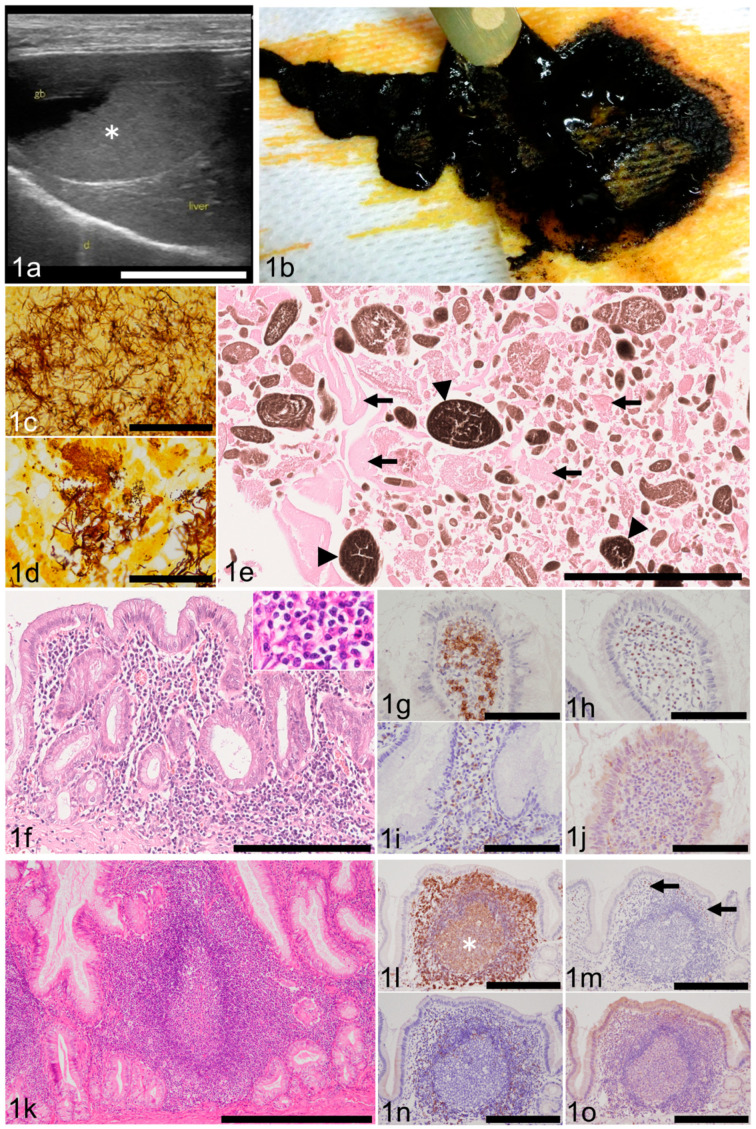
Results of gross pathological, histopathological, histochemical, and immunohistochemical investigation of canine chronic cholecystitis. (**a**) Ultrasonography revealed sandy, gravity-dependent contents interpreted as sludge (asterisk). Case 122. Bar = 2 cm. (**b**) Sludge is dark brown, viscous material. Case 12. (**c**) Filaments and rods are present within gallbladder content. Warthin–Starry (WS). Case 1. Bar = 50 µm. (**d**) Rods and coccobacilli are present within gallbladder content. WS. Case 169. Bar = 50 µm. (**e**)**.** The material ultrasonographically and grossly described as “sludge” is histologically composed of fragmented mucus (arrows) and microliths (arrowheads). Hematoxylin and eosin (HE). Case 57. Bar = 500 µm. (**f**) The lamina propria is expanded by heavy infiltrates of lymphocyte and plasma cell, interpreted as grade 2 (G2) inflammation. HE. Case 164. Bar = 200 µm. Inset: higher magnification of lymphocytes and plasma cells in the lamina propria of the gallbladder of the same dog. (**g**) Chronic cholecystitis. CD20-positive B cells predominate. Immunohistochemistry (IHC). Case 19. Bar = 125 µm. (**h**)**.** Chronic cholecystitis. A few MUM1-positive plasma cells are present. IHC. Case 19. Bar = 125 µm. (**i**) Chronic cholecystitis. A few CD3-positive T cells are present. IHC. Case 19. Bar = 125 µm. (**j**) Chronic cholecystitis. Granzyme-B-positive cells are not present. IHC. Case 19. Bar = 125 µm. (**k**) Lymphoid follicle has a prominent germinal center. HE. Case 19. Bar = 500 µm. (**l**) Many CD20-positive B cells are present in the germinal center (asterisk) and marginal zone. IHC. Case 19. Bar = 250 µm. (**m**) A few MUM1-positive plasma cells are present in the perifollicular area (arrows). IHC. Case 19. Bar = 250 µm. (**n**) Few CD3-positive T cells are present in the mantle zone and perifollicular area. IHC. Case 19. Bar = 250 µm. (**o**) Granzyme-B-positive cells are not present. IHC. Case 19. Bar = 250 µm.

The most frequently detected ultrasonographic abnormality was sludge (*n* = 105; 47.9%; Figure 1a), followed by mucocele (*n* = 34; 15.5%), gallstones (*n* = 20; 9.1%), thickened wall (*n* = 13; 5.9%), immovable contents other than mucocele (*n* = 11; 5.0%), and dilation of the common bile duct (*n* = 8; 3.6%). Cholecystitis (*n* = 3), intracystic mass(es) (*n* = 3), and rupture (*n* = 2) were rare findings. Grossly, sludge was identified as dark brown, viscous material (Figure 1b). The details of ultrasonographic abnormalities of 25 dogs were not provided by the submitters. 

### 3.3. Complete Blood Count (CBC) and Blood Chemistry

No abnormality was detected in 72 dogs. Twelve dogs had elevated white blood cell counts, while 1 dog showed mild leukopenia. Mild anemia was observed in 7 dogs. The results of CBCs of 127 dogs were not provided by the submitters. Elevated liver enzymes were noticed in 30 dogs. Other than this, elevated alkaline phosphatase (ALP, *n* = 81), alanine transaminase (ALT, *n* = 63), and C-reactive protein (CRP, *n* = 26) were relatively frequent abnormal blood chemistry results. No abnormal values were detected in 21 dogs. The results of blood chemistries for 61 dogs were not provided by the submitters.

### 3.4. Bacterial Culture and Histochemically Detected Bacteria in the Gallbladder 

Bacterial culture of the gallbladder tissue or intracystic bile was not performed in 201 dogs. Six cases, in which bacterial culture was done, revealed negative results. In one case, both aerobic and anerobic cultures were positive but bacterial identification was not pursued. Bacterial agents were detected by Giemsa stain of bile juice in one case without speciation. Results of bacterial cultures of five cases were pending at the time of sample submission but were later lost to follow-up. Giemsa and/or Warthin–Starry stains detected bacterial pathogens in 64 gallbladders (64/219; 29.2%): 41 of 60 G2 cases (68.3%), 18 of 129 G1 cases (14%), 3 of 18 G0 cases (16.7%), and 2 of 12 cases with mucosal loss (16.7%), respectively. Morphology of bacteria varied among filaments, rods, coccobacilli, or cocci (Figure 1c,d).

### 3.5. Contents of the Gallbladder

Histological examination determined that the gallbladder contents of the dogs in the study group often contained mucus (*n* = 68; 31%), microliths (*n* = 55; 25.1%), mucocele (*n* = 42; 19.1%), bile (*n* = 24; 10.9%), blood (*n* = 21; 9.5%), gallstone (*n* = 7; 3.1%), and mass (*n* = 1; 0.4%). The remaining gallbladders were devoid of contents upon histologic examination. Sludge is not listed under histological findings as it is detected by ultrasonography and not by histological methods. Content that is ultrasonographically described as sludge is a mixture of variably sized heterogeneous granular structures. These include fragmented gelatinous mucus, a brown granular substance (believed by the authors to be microliths), cell debris, and occasional bacteria (Figure 1e). As for mucocele, since some cases posed difficulty in ultrasonographic diagnosis, its prevalence was recalculated as 51 cases (23.2%) by combining the results of ultrasonography and histology. Histologically, mucocele was described as an amorphous amphophilic material filling the lumen and tightly adhering to the mucosa, which always showed marked hyperplastic change. In those dogs with gallbladder mucocele, there were eight cases with severe inflammation (G2; 8/51; 15.7%), 31 cases with mild inflammation (G1; 31/51; 60.8%), and nine cases with no inflammation (G0; 9/51; 17.6%). Necrosis or ulceration hampered examination of the mucosa in three cases (3/51; 5.9%). Gallstones were characterized as an accumulation of minerals resistant to cutting. Chemical analysis of the gallstones was not conducted in this study. 

### 3.6. Gallbladder Mucosal Inflammation (Cholecystitis)

One hundred and eighty-nine of 219 (86.3%) gallbladders had mucosal lymphoplasmacytic infiltration (Figure 1f). Within this, there were 60 G2 cases (60/219; 27.3%) and 129 G1 cases (129/219; 58.9%). Eighteen cases were judged G0 (18/219; 8.2%). Necrosis or ulceration hampered detailed examination of the mucosa in 12 dogs. Macrophages, often laden with amber to light brown lipofuscin-like pigment, were present in samples of 94 dogs. In most cases, however, the number of infiltrating macrophages was small. A few eosinophils were present in 25 G1 cases. Neutrophils were identified in seven G1 cases. By IHC, CD20-positive cells (B lymphocytes) predominated in the lamina propria (Figure 1g). A few MUM1-positive cells (plasma cells, Figure. 1h) and few CD-3 positive cells (T lymphocytes, Figure 1i) were also present. No granzyme-B-positive cells (cytotoxic T lymphocytes or natural killer cells) were detected (Figure 1j). 

### 3.7. Other Histologic Findings of the Gallbladder 

The average of gallbladder wall total thickness (GWTT) was 1071.9 µm. The median of GWTT was 859.5 µm. Normal GWTT, measured in six gallbladders of freshly (postmortem period being 3 hours or less) autopsied dogs without gallbladder-associated anamnesis or histologic evidence of mucosal lymphoplasmacytic infiltration, rarely exceeded 500 µm (data not shown). Samples of 68 dogs (68/219; 31%; n = 48 of G2, n = 20 of G1) had lymphoid follicles in the mucosa (Figure 1k). IHC determined that B lymphocytes were numerous in the germinal centers and marginal zone (Figure 1l). Plasma cells were infrequently seen in the perifollicular area (Figure 1m). T lymphocytes were scattered in the mantle zone and perifollicular area (Figure 1n). No cytotoxic T lymphocytes or natural killer cells were detected (Figure 1o). Lymphoid follicles were generally less in number and size in the G1 population than in that of G2. 

**Figure 2:**
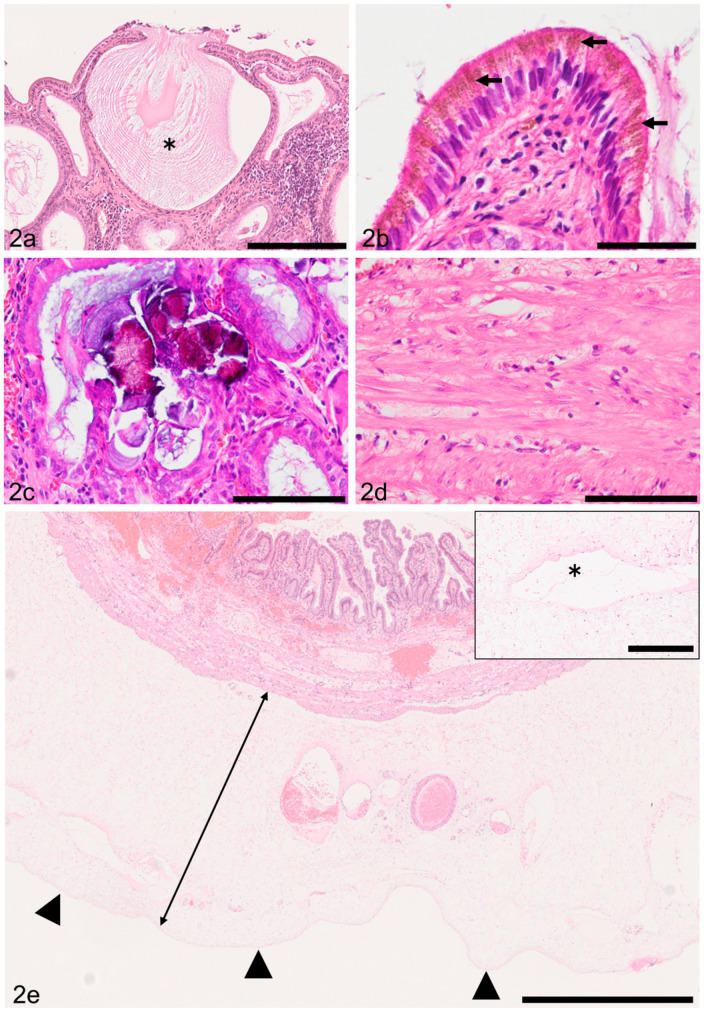
Results of histopathological investigation of canine chronic cholecystitis. (**a**) Secreted mucus (asterisk) expands the mucosal crypt. HE. Case 56. Bar = 200 µm. (**b**) Amber to light brown pigment granules resembling lipofuscin are present in the cytoplasm of mucosal epithelial cells (arrows). HE. Case 138. Bar = 50 µm. (**c**) Intracryptic mucus is replaced by basophilic to amphophilic, irregular, solid substance (mineralization/calcification of mucus). HE. Case 79. Bar = 100 µm. (**d**) Smooth myocytes show hyperplasia and hypertrophy. HE. Case 81. Bar = 100 µm. (**e**) The subserosa is widened (shown by a two-way arrow) due to edema. The serosal surface is delineated by arrowheads. HE. Case 7. Bar = 1 mm. Inset: higher magnification of the subserosa showing a dilated lymphatic (asterisk) in the gallbladder of the same dog. Bar = 200 µm.

Mucosal mucus hypersecretion was detected by histological examination as flooding mucus from the apical surface of epithelial cells in samples of 209 dogs (97.6%). Accumulation of mucus in mucosal crypts was also a frequent finding (Figure 2a). The remaining 10 dogs had ulceration or necrotic mucosa. Amber to light brown, fine granular pigment resembling lipofuscin was identified in the apical cytoplasm of epithelial cells in 164 dogs (74.8%, Figure 2b). Lymphocytic invasion within the mucosal epithelium was noticed in samples of 20 dogs. In these samples, degeneration of epithelial cells was not observed. Vacuolar change of mucosal epithelial cells was present in 6 samples (2.7%). Mineralization of mucus within mucosal crypts was detected in 14 samples (6.3%; Figure 2c). Mild to severe fibrosis in the submucosa was detected in 200 dogs (91.3%). Mild to marked thickening of the smooth muscle layer (smooth muscle thickening) was noticed in 137 dogs (62.5%). The smooth muscle thickening was characterized by an increase in the size (hypertrophy) and number (hyperplasia) of smooth myocytes (Figure 2d). Mild to severe mural inflammation, predominantly of a lymphoplasmacytic nature, was observed in 104 dogs (47.4%). In the gallbladder wall, edema (n = 116; 52.9%; Figure 2e), congestion (n = 85; 38.8%), hemorrhage (extravasation of RBCs; n = 75; 34.2%), lymphatic dilation (n = 74; 33.7%; Figure 2e), serositis (peritonitis; n = 7; 3.1%), and vascular thrombosis (n = 5; 2.2%) were also noted. Four adenomas and single cases of lymphoma, leiomyoma, and carcinoid tumor were observed in the gallbladders of our cohort.

### 3.8. Liver Inflammation

**Figure 3:**
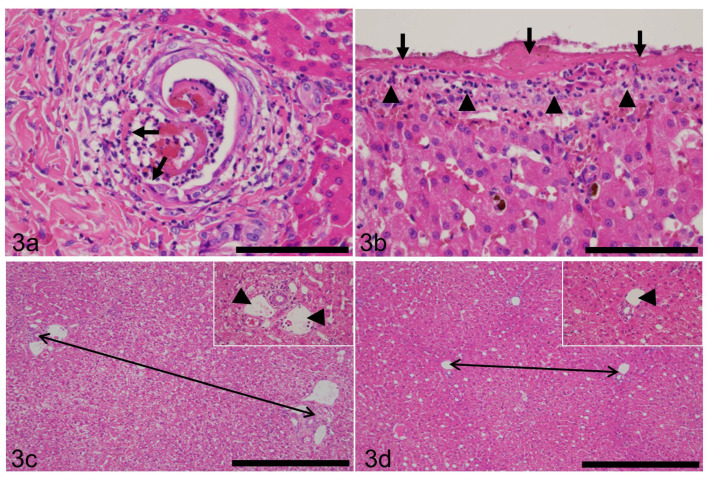
Results of histopathological investigation of the liver of the dogs with chronic cholecystitis. (**a**) Inflammatory cells, predominantly neutrophils, infiltrate the lumen and epithelium of a bile ductule (cholangitis) as well as interlobular connective tissue (pericholangitis). The epithelium of a bile ductule is eroded (arrows). HE. Case 168. Bar = 100 µm. (**b**) The hepatic capsule is covered by fibrin (arrows) and infiltrated by neutrophils (arrowheads), consistent with capsulitis/peritonitis. HE. Case 168. Bar = 100 µm. (**c**) Hepatic lobular diameter (HLD; shown by a two-way arrow) is within normal limits. Inset: higher magnification of portal tract of the same dog. Interlobular vein (arrowheads) is unremarkable. HE. Case 94. Bar = 500 µm. (**d**) HLD (two-way arrow) is significantly smaller than that of case 94 shown in (**c**). HE. Case 160. Bar = 500 µm. Inset: higher magnification of portal tract of the same dog. Interlobular vein (arrowhead) is significantly small.

Seventy-six dogs (34.7%) had inflammation in the liver. The array of inflammation included pericholangitis (*n* = 71; Figure 3a), cholangitis (n = 29; Figure 3a), hepatitis (*n* = 25), and capsulitis (peritonitis; *n* = 27; Figure 3b). Cholangitis and hepatitis tended to overlap with pericholangitis. Overall, the degree of inflammation was subjectively judged to be mild.

### 3.9. Hepatic Lobular Diameter (HLD) and Primary Portal Vein Hypoplasia (PPVH)

The average HLD in 214 liver samples was 837.8 µm, while the median was 851.5 µm (the average normal HLD value is 1042 µm based on the examination of the livers of 33 normal dogs [31]). The differences in HLD between normal and abnormal HLD are shown in Figure 3c,d. Figure 3c shows normal HLD, while Figure 3d shows decreased HLD. HLD could not be measured in five specimens due to destruction of the lobular architecture by fibrosis and the occasional coexistence of necrosis and severe inflammation. Upon histological analysis, eighty-six dogs (39.2%) were judged to have PPVH (Figure 3d).

### 3.10. Other Histologic Findings of the Liver

Hepatocellular degeneration of any sort (granular, vacuolar, or hydropic) and severeness, and/or hepatocellular necrosis of any kind (solitary, focal, or massive), degree, or topography were detected in the liver specimens of almost all dogs (*n* = 217; 99%). Among 216 dogs with hepatocellular degeneration, granular degeneration (*n* = 197; 89.9%) outnumbered glycogen/hydropic degeneration (*n* = 51; 23.2%) or fatty degeneration (*n* = 13; 5.9%). These three types of hepatocellular degeneration overlapped in specimens of some dogs. Hepatocellular necrosis was a rare finding, and its severity was generally mild. The detected neoplasm included hepatocellular carcinoma (*n* = 3), lymphoma (*n* = 2), hepatocellular adenoma (*n* = 2), and metastatic carcinoma (*n* = 1). A canalicular bile plug was found in 32 dogs (14.6%). Other relatively frequent histologic findings of the liver included nodular hyperplasia (*n* = 15), extramedullary hematopoiesis (*n* = 13), hemosiderosis (*n* = 11), and lymphatic dilation (*n* = 9). No vasculitis was detected in our liver sample pool. 

### 3.11. Statistical Analysis 

A statistical comparison between null (G0), mild (G1), and severe (G2) gallbladder mucosal inflammation was made against parameters related to signalment, ultrasonographic, gross, and histological findings (Table 2). A significant difference was observed in eight parameters among the three inflammation groups: sludge, mucocele, GWTT, bacteria, lymphoid follicle, smooth muscle thickening, edema, and liver inflammation. Of these, only smooth muscle thickening showed statistical significance in all round-robin, two-group comparisons. GWTT, bacteria, and lymphoid follicle showed a significant difference between G0 and G2, and G1 and G2, respectively. Edema showed a significant difference between G0 and G1, while sludge and liver inflammation showed a significant difference between G1 and G2. For mucocele, a significant difference was found between G0 and G2. The remaining parameters (age, breed, sex, HLD, and PPVH) were not applicable to this analysis because of no significant difference among three groups.

A statistical comparison between the presence or absence of mucocele and various parameters revealed that mucocele had a significant relationship with age, sludge, GWTT, edema, HLD, and PPVH in varying degrees (Table 3). As for sludge, edema, and PPVH, the coexistence of mucocele and each of these three parameters was significantly rare enough to be judged mutually exclusive. As for age, GWTT, and HLD, the presence of mucocele is related to larger mean values of these parameters. 

Sludge had a significant negative relationship with GWTT and liver inflammation (Table 4). Specifically, the coexistence of sludge with either GWTT or liver inflammation was significantly rare. 

## 4. Discussion

As for age distribution among patients, ultrasonographic abnormalities were detected most frequently in their middle or later age. This corresponds to previous reports [23], though the exact reason why middle-aged and senior dogs tend to suffer from gallbladder disorders is yet to be investigated. Toy or miniature breeds were overrepresented in our study. Whether this is a simple reflection of demography of dog breeds in Japan or is related to other factors needs further investigation. Neutered dogs accounted for almost 90% of the entire study population. These results differ from a previous report, in which no significant skewness was identified between the presence of sludge and the sex of the dogs [23]. On the other hand, another report that described canine cholangitis, cholangiohepatitis, and gallbladder diseases showed a similar sex composition to ours in their 54-dog cohort [19]. A similar tendency was noted in other 45-dog and 27-dog cohorts, respectively [6,18]. The overt predominance of females as well as neutered individuals in several cohorts might indicate hormonal and/or metabolic effects on gallbladder disorders and indicates the need for additional case-control studies to know the basis for this result. 

Ultrasonographic abnormalities of the gallbladder have been vigorously investigated [6,24,30,32]. Clinical planning of veterinary clinicians for gallbladder mucocele seems to be well standardized and is dependent on specific ultrasonographic features detailing the disease stage and concurrent clinical information [30]. Though sludge in the gallbladders of dogs has been regarded as an incidental finding without need for treatment [4], 9 out of 24 dogs with mobile sludge or precipitate experienced gallbladder rupture in a previous study [6]. Furthermore, a recent report by Saunders et al. discusses the merit of early cholecystectomy in dogs with gallbladder sludge, emphasizing a possible relationship of sludge and mucocele and minimizing a chance of complication during cholecystectomy [26]. It is premature to make a conclusive statement on the best clinical approach for canine gallbladder sludge for the time being. In addition, as DeMonaco and colleagues [20] and Secchi’s group [23] have reported, a mere one-year follow-up of a spontaneous course of biliary sludge may not be long enough to fully understand its true nature as disease.

In prior research on animal gallbladders, detection of intralesional bacteria was attempted on various specimens using a variety of methods such as cholecystocentesis, direct sampling of the liver or gallbladder tissue during laparotomy/laparoscopy, or histochemistry (Gram stain) on archived histologic specimens [12,18]. Fluorescence in situ hybridization has also been demonstrated to be a sensitive method to detect bacteria in mucocele specimens [33]. In our study, culture of the gallbladder tissue or intracystic bile was seldom performed by the attending veterinarians, likely because of financial issues. We chose Giemsa and Warthin–Starry stains because they are cost-effective for the detection of bacteria in histology sections. The hurdle we encountered, however, was frequent leakage and loss of especially liquid gallbladder contents during tissue trimming. In addition, we were unable to speciate the pathogens by histomorphological observation alone. The past reports on bacterial isolation from dogs with hepatobiliary diseases included intestinal bacteria such as *Escherichia coli*, *Enterococcus* sp., *Bacteroides* sp., and *Clostridium* sp. [29]. Whether these bacteria are involved with initiation and progression of cholecystitis, however, has long been controversial. Since we have not been able to track periodic changes of the gallbladder except by imaging studies, reproducible cholecystitis models and new methods to trace histological/cytological changes in gallbladders are needed. Future investigation using primary cell culture of the canine gallbladder mucosal epithelial cells, such as that developed by Oda and colleagues [34], would increase our understanding on the response of gallbladder tissue against infectious agents.

Gallbladder contents can be roughly divided into two categories: mucocele and non-mucocele. While mucocele has been a target of a series of vigorous investigations, [11,21,30,32,35,36] non-mucocele contents have gained little attention in veterinary medicine. Sludge in our cohort was histologically composed of fragmented gelatinous mucus, microliths, cell debris, and bacteria. Mucus that we microscopically detected in a majority of specimens resembled mucocele in their staining features (amphophilic to pale basophilic) and texture (homogenous and amorphous). This was consistent with previous work by Mizutani and colleagues, who clarified by infrared spectroscopy that both mucocele and sludge were equivalent to swine mucin in their chemical properties [12]. Microliths, on the other hand, are aggregates of a brown, soft, granular substance interpreted as condensed bile in our study. Microliths grossly look very much like brown-pigmented human gallstones though they are typically formed in the bile ducts, not in the gallbladder as seen in our canine cohort [37,38]. Human brown-pigmented gallstone is associated with biliary infection, which is likely the case in our study [37]. Further investigation is necessary to determine the nature of microliths and if these are actually premature gallstones. 

Mucocele, in our study, frequently occurred along with mild cholecystitis. This result is consistent with the findings in a previous work by Aguirre and colleagues [1]. As Mizutani’s group pointed out, a sequential development from sludge-forming gallbladder disease to mucocele is a possible scenario [12]. Tsukagoshi and colleagues also reported that postprandial gallbladder emptying was significantly reduced in those dogs with mobile/immobile sludge or mucocele when compared to control dogs without sludge or mucocele, suggesting that biliary stasis is an important pathologic basis for gallbladder diseases in general [24]. In order to confirm that there is a transition from sludge-forming cholecystitis to mucocele, sequential evaluation on changes occurring in the gallbladder tissue in vivo, which does not seem feasible now, would be necessary by novel in vitro techniques. 

A comprehensive and detailed description on cholecystitis in canine gallbladders has not been reported, except for the work by Lawrence and colleagues describing as few as six gallbladders [39], as well as the report of Viljoen’s group on just 14 gallbladders [25]. The present report is the first among the similar kinds that board-certified veterinary pathologists have conducted the comprehensive histomorphological evaluation of diseased canine gallbladders. In humans, on the other hand, histopathology of cholecystitis has been well documented [37]. For example, in humans, cholecystitis is divided into acute and chronic forms [37]. Each form includes calculous (gallstone-laden) and acalculous (no gallstone) cholecystitis [37]. Human chronic “acalculous” cholecystitis has subclasses such as lymphoeosinophilic, eosinophilic, granulomatous, diffuse lymphoplasmacytic, and lymphocytic cholecystitis [37]. Some chronic human “calculous” cholecystitis cases have minor components of plasma cells, eosinophils, macrophages, and neutrophils [37]. “Calculous” versus “acalculous” nomenclature in veterinary medicine needs clarification to determine whether microliths are precursors of gallstones. Canine microliths grossly and microscopically resemble brown-pigmented gallstones of humans, but we need further physical and chemical research to clear this point. Fixing a nomenclature and disease classification is important to facilitate crosstalk between medical and veterinary medical experts as well as to support clinical decisions of veterinary practitioners.

Lymphoid follicles have not previously been reported in canine gallbladders. Prolonged antigenic stimulation seems to predispose this, but the precise etiology is yet to be investigated. In humans, follicular cholecystitis (FC) has been diagnosed by detecting three or more distinct lymphoid follicles per centimeter, and is associated with older age [40]. Saka et al. reported that FC did not seem to be associated with autoimmunity, lymphoma, or obstructive gallbladder diseases in an investigation of 2550 human cholecystectomy specimens, in which there were only five samples fulfilling the diagnostic criteria of FC [40]. We may need to refrain from using the term FC for canine gallbladders until there is a consensus on terminology among experts.

Contrary to the frequent isolation/detection of bacterial pathogens, neutrophilic inflammation was not frequently identified in canine gallbladders in our study and in the studies of others [25,39]. This finding corresponded with the previous report on porcine chronic cholecystitis, in which lymphoplasmacytic inflammation was associated with the frequent detection of bacterial bacilli by use of the Warthin–Starry stain [41]. Contrary to this, neutrophilic inflammation was much more prevalent in the livers of those dogs suffering from gallbladder diseases [19]. Gallbladders with prominent neutrophilic infiltration in our canine population, though their number was low, seemed to represent acute-on-chronic inflammation, in which peracute secondary bacterial inflammation overlaid a pre-existing chronic lymphoplasmacytic cholecystitis.

IHC revealed the composition of lymphoid cells in 12 G2 lesions of our cohort. B lymphocytes predominated in the lamina propria, and a few plasma cells and fewer T lymphocytes were also seen. Granzyme-B-positive cells, suggestive of cytotoxic T lymphocyte or natural killer cell origin, were not detected in our study. Taken together with the detection of lymphoid follicles in 68 tissues (48 of G2; 20 of G1), there likely was a long-standing stimulation to the mucosal tissue by as yet unknown antigenic elements such as bacterial or bile-related constituents. The frequent detection of plasma cells around follicles was intriguing. This suggests increased production of immunoglobulin in this tissue. Since gallbladder mucin and immunoglobulin G have shown to accelerate nucleation in the development of gallstones, B-cell-dominant inflammation with plasma cells might be a research target to understand the mechanism of sludge/gallstone formation [38]. Future studies from the standpoint of immunology are definitely needed.

A detailed histological evaluation of the thickened gallbladder wall has not been performed though it is a common abnormality observed in ultrasonographic examination of diseased gallbladders [6,18,32]. Crews and colleagues documented intramural necrosis, hemorrhage, vascular thrombosis, inflammation, fibrosis, and mucosal hyperplasia in their 45-dog cohort; however, all of their subjects had gallbladder rupture, unlike our cohort [6]. Our investigation was the first to describe the entire gallbladder wall with great detail, including measurement of mucosal thickness (unpublished data) and GWTT. The average (1071.9 µm) and median (859.5 µm) of GWTT obtained by our study was larger than that of normal dogs, in which GWTT rarely exceeds 500 µm (observation in routine histopathology by the authors). Contrary to previous reports, thickening of the gallbladder wall in our chronic cholecystitis cases was caused by mucosal hyperplasia, smooth muscle thickening, fibrosis, edema, congestion, lymphatic dilation, and hemorrhage. Smooth muscle thickening was associated with myocytic hyperplasia/hypertrophy, suggesting increased mechanical burden during gallbladder emptying, abnormal nerval inputs, or an effect of growth factor(s). Gallbladder wall edema has been reported in canine patients with pulmonary hypertension or anaphylaxis, implying an effect of increased static pressure or increased vascular wall permeability [42,43]. The underlying pathogenesis for cholecyst wall edema, in our cases, likely differs in each case and may represent concurrent inflammatory status and/or vascular wall physiopathology. Intramural fibrosis of the gallbladders in our cohort was likely related to tissue repair during the chronic inflammatory process. 

Intracytoplasmic accumulation of amber to light brown, fine granular pigments has not previously been reported in canine gallbladders. Gilloteaux and others conducted ultrastructural studies on chronic human cholecystitis and found osmiophilic lipofuscin-like bodies and lipomucosomes (a fusion of lipid deposits and mucus-containing vesicles in cholecystocytes) [44]. These intracellular, as well as extracellular, structures led these researchers to hypothesize that intracellular components are released outside the cell by the detergent-like action of bile on cholecystocytic membranes, which then become aggregated to form biliary sludge [45].

Mineralization of mucus, typically within mucosal crypts, as in our cases, has not been described previously in canine gallbladders to the authors’ knowledge. There was a microscopically detectable transition between bland intracryptic mucus and mineralized crystals; therefore, a calcium-binding property of mucus is speculated. The detailed mechanism behind it, however, should be clarified through investigations such as that of Imano et al., who identified osteopontin, a calcium-binding protein, in gallbladder epithelial cells and intralesional macrophages by IHC [46]. 

Seventy-six dogs in our cohort had hepatic inflammation of a varied nature and degree, which is consistent with previous reports [18,39]. When a patient presents with bacterial hepatitis, it must be determined whether it is caused by ascending or hematogenous bacterial entry. When it comes to cholecystitis, we also have to determine whether the introduction of bacteria occurs by ascending or hematogenous entry. For cholecystitis of animals, the frequent detection of alimentary bacterial species supports the ascending infection theory [6,12,18,19,28,29]. Differences in pathologic features of gallbladder diseases between cats and dogs should also be taken into consideration [28,47]. In particular, bacteremia, not bactibilia, should be closely examined in future studies to clarify the entry route and exact roles of alimentary pathogens involving hepatobiliary diseases. 

Hepatic lobular diameter (HLD) is an objective indicator to assess microhepatica, pathognomonic findings for a hepatic vascular anomaly such as PPVH and portosystemic shunts in dogs [31]. The reason we measured HLD in our study was because we hypothesized that a decreased mass of liver (decreased number of total hepatocyte) may lead to a decreased net volume of a hepatocyte-derived, yet-unknown protective element for a gallbladder mucosal epithelial cell. If we can correlate PPVH (diagnosed by a combination of “decreased” HLD and other already-mentioned histologic findings [31]) with gallbladder abnormality, the liver, as a producer of various physiologic elements, can be an important and novel target for further research. Our results, however, did not show a statistically significant relationship among decreased HLD, mucocele, and sludge. This result indicates that PPVH, a frequently diagnosed nonlethal condition in miniature or small breed dogs, is less likely a contributor for the development of gallbladder disorders. 

Hepatocellular degeneration was detected in almost all dogs in this study, supporting the frequent previous detection of increased serum liver enzyme activities. Severity of degeneration, however, was typically mild among our subjects. Those dogs with more severe hepatocellular degeneration often had concurrent significant inflammation in portal tracts. In previous reports, hepatocellular degeneration of varying degree and nature was also detected in canine patients with gallbladder diseases, but a precise description of each liver component (portal tract, parenchyma, capsule, and so on) was not available [1,16,19,39].

The cause of chronic cholecystitis is an enigma. There have been a handful of opinions with/without supportive evidence regarding this disease. Frequent isolation/detection of alimentary bacterial species from gallbladder contents/tissues has led to a deeply entrenched notion that cholecystitis is most likely derived from bacterial infection. It may be true, but we have not been able to reproduce cholecystitis by artificial bacterial infection alone. Research by Kaminski et al., using feline subjects, suggested the possible role of arachidonic acid metabolites on the development of cholecystitis, but it has yet to be proven that this applies to cholecystitis in other animal species [48]. Kakimoto and colleagues have attributed canine gallbladder diseases to altered bile acid composition [22]. Ultrastructural work by Gilloteaux et al. suggested a possible involvement of altered lipid metabolism, which causes a subcellular accumulation of lipid deposits and a resultant sloughing of cholecystocytes (gallbladder mucosal epithelial cells), leading to the formation of bile sludge [45]. Hence, at this point, it would be safe to say that chronic cholecystitis seems to be a disease of multifactorial etiology. Similarly, there has been much speculation on the factors contributing to gallbladder mucocele, which is beyond the scope of our study [8,9,11,49]. Kesimer and colleagues performed an extensive analysis of canine gallbladder mucus, including the physical, chemical, and functional features of mucus, in their study of mucus hypersecretion in canine gallbladders [35]. This would be a powerful approach to the study of chronic cholecystitis pathobiology as well.

Lastly, our statistical analysis was designed to determine whether null (G0), mild (G1), or severe (G2) gallbladder inflammation was related to any ultrasonographic or clinicopathological parameters. Those parameters showing statistical significance between G0 vs. G1 or G0 vs. G2 were judged to be useful in differentiating between an “inflamed gallbladder” and an “uninflamed gallbladder”. Such parameters included mucocele, GWTT, bacteria, lymphoid follicles, smooth muscle thickening, and edema. Among these features, smooth muscle thickening and edema could be a more sensitive indicator of gallbladder inflammation than the rest because these two parameters showed statistical significance even in the pair of G0 vs. G1, while others did so only in G0 vs. G2 pairing. On the other hand, those parameters showing statistical significance between G1 vs. G2 were judged to be of some use in predicting the “severity” of cholecystitis. Parameters associated with severity were sludge, GWTT, bacteria, lymphoid follicle, smooth muscle thickening, and liver inflammation. We also examined the relationship between sludge or mucocele and various clinicopathological parameters. The results showed that mucocele was mutually exclusive with sludge, edema, and PPVH. On the other hand, mucocele was related to increasing values of age, GWTT, and HLD. Most of these results are intuitive and supported by histological features. PPVH does not seem to predispose the patient to mucocele formation, so mucocele is likely a disease independent of liver abnormality. As for sludge, our analysis revealed a mutual exclusion of sludge with GWTT or liver inflammation. Therefore, gallbladders with normal wall thickness could have undergone a pathologic process. In addition, sludge can be caused by a process indifferent to the inflammatory condition of the liver. All these findings based on statistics, however, should be interpreted and extrapolated to real-world settings with caution until additional evidence is collected.

Our study had some shortcomings. First, the majority of ultrasonography was not performed by board-certified radiologists, so comparing our results with those of other researchers may yield some discrepancy. However, since our study objective was to pathologically investigate ultrasonographically “abnormal” gallbladders, and we histologically reclassified mucocele versus non-mucocele specimens, the results of our study should be regarded as reasonable enough to reach a conclusion. The second was a partial lack of clinical and clinicopathological information due to our retrospective approach. This impaired the completeness of evaluation on relationships among various gallbladder diseases and clinical/clinicopathological parameters. Thirdly, we could not speciate bacteria in the gallbladder lumen because we relied on cost-effective histochemistry to evaluate bacterial pathogens. Thinking of a large number of cases of positive bacterial detection (more than 60 cases in total), however, an attempt to speciate all the pathogens through molecular methodology (PCR or next-generation sequencing) was beyond our capacity. In the future, we or other investigators are encouraged to plan accordingly to take care of these matters.

## 5. Conclusions

In summary, our study on gallbladders and livers of 219 client-owned dogs reported previously unrecorded findings, especially detailed descriptions of chronic cholecystitis of dogs. Our results indicate that sludge in canine gallbladders could be a sign of an insidious inflammatory process of yet undetermined etiopathology. A relationship among sludge, mucocele, and chronic cholecystitis likely exists, but further evidence is needed to support that conclusion. Frequent detection of lymphoplasmacytic inflammation and lymphoid follicles suggests long-standing antigenic stimulation by an unknown, but likely bacterial, agent. Our statistical analysis provides information for the evaluation and interpretation of multiple clinicopathological, ultrasonographic, and histomorphological data. Of note, the pathogenesis of sludge and mucocele seems independent of liver abnormalities. Future research on the canine gallbladder should encompass the perspective of inter-organ relationships, and a multidisciplinary approach would yield results that most accurately determine the role of this intriguing organ in health and disease. 

## Figures and Tables

**Table 1:** Primary antibodies used for immunohistochemistry.

Antibody to	Host	Type	Dilution	Source	Catalogue Number
CD3	Mouse	Monoclonal	1:50	Abcam, Cambridge, UK	ab17143
CD20	Rabbit	Monoclonal	1:200	Abcam, Cambridge, UK	ab64088
MUM1	Rabbit	Monoclonal	1:500	Abcam, Cambridge, UK	ab133590
Granzyme B	Rabbit	Polyclonal	1:50	Abcam, Cambridge, UK	ab4059

**Table 2:** Results of statistical comparison between null inflammation (G0), mild inflammation (G1), and severe inflammation (G2) against parameters related to signalment, ultrasonographic, and histologic findings.

	*p*-Value			
Parameter	G0 vs. G1 vs. G2	G0 vs. G1	G0 vs. G2	G1 vs. G2
Age	0.113	N/A	N/A	N/A
Breed	0.061	N/A	N/A	N/A
Sex	0.727	N/A	N/A	N/A
Sludge	0.025 *	0.208	1.000	0.013 **
Mucocele	0.005 *	0.044	0.002 **	0.122
GWTT ^a^	0.000 *	1.000	0.000 **	0.000 **
Bacteria	0.000 *	0.724	0.000 **	0.000 **
Lymphoid follicle	0.000 *	0.134	0.000 **	0.000 **
Smooth muscle thickening	0.000 *	0.011 **	0.000 **	0.000 **
Edema	0.021 *	0.010 **	0.106	0.267
Liver inflammation	0.000 *	0.064	0.102	0.000 **
HLD ^b^	0.566	N/A	N/A	N/A
PPVH ^c^	0.304	N/A	N/A	N/A

* *p* < 0.05. ** *p* < 0.017 (note: the significance level of two-group comparison in multiple comparison using Bonferroni method is α = 0.05/3 = 0.017). ^a^ GWTT = gallbladder wall total thickness; ^b^ HLD = hepatic lobular diameter; ^c^ PPVH = primary portal vein hypoplasia.

**Table 3:** Results of statistical comparison between presence/absence of mucocele and other parameters regarding signalment, ultrasonographic, and histologic findings.

Parameter	Coefficient of Association	*p*-Value
Age	0.420 ^(1)^	0.010 *
Breed	0.282	0.788
Sex	0.069	0.787
Sludge	0.183	0.010 *
GWTT ^a^	0.160 ^(2)^	0.018 *
Bacteria	0.116	0.113
Lymphoid follicle	0.066	0.389
Smooth muscle thickening	0.109	0.137
Edema	0.260	0.000 *
Liver inflammation	0.120	0.093
HLD ^b^	0.515 ^(1)^	0.002 *
PPVH ^c^	0.200	0.003 *

* *p* < 0.05. ^a^ GWTT = gallbladder wall total thickness; ^b^ HLD = hepatic lobular diameter; ^c^ PPVH = primary portal vein hypoplasia. ^(1)^ Cohen’s d; ^(2)^ point-biserial correlation coefficient; others, Cramér’s V.

**Table 4:** Results of statistical comparison between presence/absence of sludge and other parameters regarding signalment, ultrasonographic, and histologic findings.

Parameter	Coefficient of Association	*p*-Value
Age	0.087 ^(1)^	0.518
Breed	0.326	0.443
Sex	0.094	0.583
GWTT ^a^	0.274 ^(2)^	0.000 *
Bacteria	0.114	0.103
Lymphoid follicle	0.091	0.191
Smooth muscle thickening	0.070	0.330
Edema	0.062	0.417
Liver inflammation	0.277	0.000 *
HLD ^b^	0.122 ^(1)^	0.373
PPVH ^c^	0.052	0.490

* *p* < 0.05. ^a^ GWTT = gallbladder wall total thickness; ^b^ HLD = hepatic lobular diameter; ^c^ PPVH = primary portal vein hypoplasia. ^(1)^ Cohen’s d; ^(2)^ point-biserial correlation coefficient; others, Cramér’s V.

## Data Availability

The data presented in this study are available on request from the corresponding author. Caution should be exercised, however, that the whole-slide histology images (so-called virtual slides) are too heavy to transmit via email or other electronic transmission modalities. Requesters for these data should provide a HDD of 3TB or larger.

## Supplementary Materials

The following is available online at https://www.mdpi.com/article/10.3390/ani11113324/s1, Supplementary Table S1: Signalment and results of clinicopathological investigation of 219 dogs.
