# Supplementary material for: Chronic Cholecystitis of Dogs: Clinicopathologic Features and Relationship with Liver
Source: Animals (Basel). 2021 Nov 21;11(11):3324. doi: 10.3390/ani11113324 (PMC8614729; doi:10.3390/ani11113324)
Supplement: Supplementary file 1 [file animals-11-03324-s001.zip › animals-1445841-supplementary.pdf]

Supplementary Table S1. Signalment and results of clinicopathological investigation of 219 dogs.

Number	Age[a]	Breed	Sex	Sludge	Mucocele	G2[b]	G1[c]	G0[d]	GWTT[e]	Bacteria	Lymphoid follicle	Edema	Smooth muscle thickening	Liver inflammation	HLD[f]	PPVH[g]
1	156	sheltie	CM[h]		P[i]	P			6930	P	P		P		874	
2	168	mini schnauzer	M[j]		P		P		1660				P	P	809	
3	92	toy poodle	CM				P		1550			P			823	
4	135	pomeranian	CM	P					297					P	368	P
5	133	shih tzu	CM	P			P		1170			P			782	P
6	72	chihuahua	CM	P	P		P		1010						1146	
7	68	shiba	F[k]	P		P			1990			P	P	P	547	P
8	72	mini dax	CM			P			1450	P	P	P	P	P	1056	
9	83	beagle	SF[l]	P			P		922			P			825	
10	145	chihuahua	SF	P		P			1360	P	P	P	P		968	
11	115	jack russell terrier	SF	P			P		721			P			786	P
12	142	toy poodle	SF	P			P		1020			P			932	
13	145	mini dax	SF	P		P			894		P		P		949	
14	118	papillon	SF				P		1060			P			818	
15	110	jack russell terrier	SF	P			P		704		P		P		896	
16	155	shih tzu	SF	P	P		P		1740			P	P	P	851	
17	84	chihuahua	CM	P			P		725			P			825	
18	132	sheltie	SF			P			916		P		P	P	1052	
19	98	toy poodle	SF			P			1670	P	P		P		1256	
20	149	welsh corgi	M	P			P		835	P		P			1099	
21	149	mini dax	SF			P			912		P	P	P		711	P
22	114	mini schnauzer	SF	P			P		1320			P	P		1081	
23	179	toy poodle	CM				P		556			P			797	P
24	172	chihuahua	SF		P	P			1510	P	P		P	P	971	
25	147	mini dax	CM		P	P			2010	P			P	P	1450	
26	119	toy poodle	CM	P	P		P		753			P	P	P	1085	
27	151	mini dax	SF		P		P		1190			P	P		1201	
28	168	american cocker spaniel	SF		P			P	8770	P				P	1210	
29	111	mini dax	SF			P			1160			P	P	P	751	P
30	116	labrador	F				P		1780			P	P	P	986	
31	121	pug	SF						1750					P	1143	
32	140	chihuahua	CM		P			P	663					P	1133	
33	173	papillon	SF		P		P		1000			P	P		724	P
34	122	mini dax	CM	P			P		860		P	P	P		912	
35	30	french bull	M	P		P			1780		P	P	P		989	
36	104	toy poodle	SF				P		994			P	P		861	
37	115	mini schnauzer	SF	P			P		445		P			P	833	
38	127	shiba	SF	P			P		917			P	P		765	P
39	180	mini dax	SF		P		P		831		P		P		1052	
40	86	mix	SF			P			1140	P	P		P		892	
41	148	toy poodle	CM		P			P	643						996	
42	149	mini dax	SF	P	P		P		586						675	P
43	172	toy poodle	CM	P			P		434						519	P
44	181	mix	CM		P			P	2040						890	
45	95	toy poodle	CM	P			P		370		P				1075	
46	189	mini dax	CM		P		P		5190						874	
47	103	toy poodle	M		P			P	1760						815	P
48	36	toy poodle	F	P			P		613			P			964	
49	96	papillon	SF		P		P		578		P		P	P	709	P
50	50	mini schnauzer	F	P			P		307		P	P			771	P
51	132	mini dax	SF	P		P			667		P		P	P	780	P
52	140	mini dax	M			P			1450	P	P	P	P	P	898	
53	78	toy poodle	SF		P		P		553				P	P	923	
54	155	sheltie	SF		P		P		717		P		P		969	
55	146	french bull	SF	P			P		536			P	P		861	
56	124	yorkshire terrier	SF				P		840			P	P		750	P
57	150	mini dax	SF				P		203			P			904	
58	131	toy poodle	M				P		633	P			P	P	626	P
59	21	toy poodle	M	P			P		850		P	P	P		807	
60	143	toy poodle	CM	P			P		604			P			744	P
61	120	yorkshire terrier	SF	P		P			834	P	P	P	P	P	677	P
62	75	chihuahua	CM						762					P	753	P
63	146	papillon	CM	P		P			809	P	P	P	P	P	680	P
64	152	mini dax	SF	P	P		P		796		P				883	
65	144	mini dax	SF				P		825			P	P	P	716	P
66	119	yorkshire terrier	CM	P		P			1020		P	P	P		809	
67	100	mini schnauzer	CM				P		848			P	P	P	847	
68	110	toy poodle	CM	P			P		401			P	P		745	P
69	161	chihuahua	CM			P			1540		P		P	P	896	
70	112	toy poodle	CM				P		966			P	P		783	P
71	192	shiba	F	P	P		P		1320	P		P		P	797	
72	113	mini dax	CM				P		493			P			1403	
73	162	sheltie	CM			P			1450	P		P	P	P	698	P
74	96	chihuahua	CM	P			P		644	P		P	P		898	
75	132	pomeranian	CM	P			P		481	P					838	
76	117	toy poodle	SF				P		392			P	P		959	
77	131	toy poodle	CM	P	P		P		524	P			P		1116	
78	170	mini dax	CM			P			3800				P	P	825	
79	172	mini dax	CM			P			1340	P	P	P	P		913	
80	127	chihuahua	CM				P		848		P	P	P		712	
81	86	mix	SF			P			1650		P	P	P	P	944	
82	175	toy poodle	CM	P		P			2070		P	P	P	P	1103	
83	108	toy poodle	CM	P	P		P		460			P	P		793	
84	99	chihuahua	SF				P		558			P			1146	
85	129	pug	SF				P		1050			P	P		763	P
86	119	papillon	SF	P			P		960			P	P		678	P
87	154	chihuahua	SF	P					664					P	895	
88	120	mini dax	CM	P		P			2270	P	P		P	P	701	P
89	163	american cocker spaniel	SF		P		P		1670			P	P	P	826	
90	158	Chihuahua	CM			P			2610	P	P		P	P	NA	
91	156	mini dax	SF				P		949	P		P	P		608	P
92	87	french bull	CM	P				P	523			P	P		785	P
93	142	papillon	M	P			P		856			P	P	P	944	
94	108	mini schnauzer	CM		P				381					P	1038	
95	140	french bull	SF	P			P		627			P			715	P
96	109	toy poodle	CM	P			P		568	P		P	P		950	
97	152	mini dax	CM				P		1050			P	P		1103	
98	149	mini dax	SF	P			P		787				P		1163	

99	157	mini dax	SF	P		P			1430	P			P	P	1061	
100	157	shiba	M	P			P		560			P			992	
101	141	mini schnauzer	SF	P	P		P		462						1031	
102	170	shih tzu	SF			P			1420	P	P	P	P	P	814	
103	127	shiba	SF	P			P		562			P	P		1073	
104	131	shiba	CM		P		P		1170					P	953	
105	156	chihuahua	SF		P		P		1190			P	P	P	937	
106	152	american cocker spaniel	CM	P		P			1230			P	P		860	
107	203	chihuahua	M			P			1480	P	P		P	P	729	
108	156	pomeranian	SF	P		P			1750	P	P		P	P	732	P
109	55	toy poodle	SF	P				P	322			P			925	
110	138	pomeranian	CM	P		P			1650	P	P		P	P	602	P
111	178	maltese	SF			P			810	P	P		P	P	519	P
112	119	mini dax	SF	P				P	407			P	P		723	P
113	103	pomeranian	CM	P			P		760	P			P		666	P
114	187	maltese	SF	P			P		498				P		656	P
115	112	maltese	SF				P		1190				P	P	683	P
116	98	chihuahua	SF				P		736				P		663	P
117	96	toy poodle	SF	P			P		935		P	P			734	P
118	136	shiba	M		P				1470	P		P		P	785	P
119	138	toy poodle	SF	P			P		1510	P	P	P	P		866	
120	120	chihuahua	CM	P			P		405				P		770	
121	173	shih tzu	CM				P		981	P		P	P		691	P
122	96	shiba	SF	P			P		655				P		724	P
123	95	yorkshire terrier	SF		P		P		489				P		767	
124	132	G shepherd	M			P			2130	P	P		P		682	P
125	69	mix	CM						1640	P					993	
126	102	yorkshire terrier	F				P		1300	P				P	876	
127	120	mix	CM				P		840			P	P		1026	
128	166	shiba	CM	P		P			1360	P			P		733	P
129	134	pug	SF	P			P		897				P		867	
130	119	yorkshire terrier	SF		P	P			937	P	P	P	P		800	
131	119	pomeranian	SF		P				1040				P		715	
132	127	mix	CM	P			P		627				P		823	
133	66	mini dax	CM			P			1020	P	P	P	P		760	P
134	165	chihuahua	SF	P					926				P		735	P
135	164	mix	SF			P			1440	P	P	P	P	P	717	P
136	144	shiba	SF			P			2280			P	P		621	P
137	109	WHWT	CM				P		1220			P	P	P	NA	
138	121	chihuahua	CM				P		894			P	P		896	
139	128	mini dax	M	P		P			1880	P	P	P	P	P	632	P
140	100	pomeranian	CM						1560						832	
141	107	mini dax	F	P			P		1120	P	P	P	P		687	P
142	55	mix	SF				P		271			P			924	
143	62	toy poodle	CM				P		484			P			1034	
144	154	mini dax	SF	P			P		715		P	P	P		881	
145	74	chihuahua	SF	P			P		334			P			883	
146	171	mini dax	SF			P			699	P	P	P		P	706	P
147	171	mini dax	M				P		407	P			P		987	
148	150	toy poodle	SF					P	1100	P			P	P	892	
149	74	toy poodle	CM	P			P		357	P		P	P		782	P
150	42	toy poodle	SF	P				P	189	P					789	P
151	145	toy poodle	SF	P			P		814						898	
152	147	toy poodle	SF				P		461			P	P		582	P
153	85	jack russell terrier	SF	P			P		701				P		652	P
154	109	pomeranian	CM			P			795	P	P		P		837	
155	150	maltese	SF	P	P			P	524					P	1101	
156	168	mini dax	SF			P			1440	P	P	P	P	P	610	P
157	148	papillon	SF	P			P		1100			P	P		909	
158	49	french bull	SF			P			1750	P	P	P	P	P	882	
159	168	mini dax	CM	P			P		643			P			728	P
160	123	chihuahua	SF	P	P		P		1550			P			501	P
161	136	mini schnauzer	SF	P		P			1080	P	P		P	P	912	
162	146	mix	SF	P			P		534			P			740	P
163	168	sheltie	SF				P		544			P		P	593	P
164	193	papillon	CM			P			939	P	P	P	P	P	568	P
165	102	G ret	SF	P	P		P		1160			P		P	767	P
166	48	toy poodle	CM				P		744	P		P			984	
167	137	sheltie	CM	P	P		P		735				P		1115	
168	112	mini dax	SF			P			1180		P	P	P	P	748	P
169	107	yorkshire terrier	SF			P			881	P	P		P		622	P
170	142	mini dax	SF		P	P			996		P		P	P	737	P
171	132	mini dax	SF				P		666			P			714	P
172	91	dax	CM	P		P			1490			P	P		1118	
173	91	welsh corgi	SF	P			P		1720			P	P		784	P
174	183	pug	SF		P		P		1090				P	P	994	
175	149	mini dax	CM			P			1610	P	P		P	P	854	
176	70	toy poodle	CM				P		318	P	P	P		P	655	P
177	152	mini schnauzer	CM	P			P		758			P	P		670	P
178	146	mini dax	SF				P		972			P	P	P	878	
179	150	chihuahua	CM	P	P		P		1700			P			833	
180	161	beagle	CM	P				P	407			P			701	P
181	162	chihuahua	SF	P	P	P			1680	P	P		P		859	
182	127	welsh corgi	SF		P	P			1700	P	P		P	P	678	P
183	104	chihuahua	SF			P			1260	P		P	P	P	574	P
184	24	norfolk ter	SF					P	1260			P	P		645	P
185	192	mini dax	SF	P			P		629	P					576	P
186	164	mix	SF		P		P		583						819	
187	122	mix	SF	P			P		762			P			749	P
188	168	toy poodle	F		P			P	846					P	875	
189	175	chihuahua	SF	P			P		425						796	
190	61	toy poodle	CM				P		492						857	
191	88	yorkshire terrier	CM	P	P		P		494			P			845	
192	161	toy poodle	SF	P			P		206			P		P	878	
193	123	american cocker spaniel	SF	P			P		683				P		674	P
194	107	mix	CM			P			2090	P	P		P	P	785	P
195	145	mini schnauzer	SF	P			P		267		P				558	P
196	86	toy poodle	SF	P			P		702			P	P		882	
197	102	french bull	SF				P		708			P			734	P
198	156	papillon	SF	P			P		887				P		873	
199	146	maltese	SF	P		P			1360		P	P	P	P	846	

200	108	toy poodle	SF						570						NA	
201	148	mix	M		P	P			1750		P	P	P		NA	
202	72	shiba	F		P			P	909					P	706	P
203	174	mini dax	CM			P			1560	P	P	P	P	P	879	
204	189	mini dax	SF	P		P			789	P	P		P		622	P
205	132	shih tzu	SF		P			P	2450					P	614	P
206	145	mini dax	M				P		557						836	
207	60	chihuahua	M						755					P	947	
208	132	shih tzu	CM				P		539						748	P
209	101	toy poodle	SF		P		P		1100		P				892	
210	175	chihuahua	CM					P	105						742	P
211	36	mix	SF	P				P	212						828	
212	126	pomeranian	M		P		P		2710		P		P		932	
213	152	mini dax	SF	P		P			782	P			P		1025	
214	111	mini dax	SF				P		859		P	P	P		867	
215	144	chihuahua	CM	P			P		533						880	
216	137	toy poodle	CM	P			P		788				P		NA	
217	120	mix	CM	P			P		639				P		714	P
218	144	mini schnauzer	CM		P		P		481				P		989	
219	132	toy poodle	SF	P			P		269						805	

[a]Months, [b]severe gallbladder mucosal inflammation, [c]mild gallbladder mucosal inflammation, [d]no gallbladder mucosal inflammation, [e]gallbladder wall total thickness, [f]hepatic lobular diameter, [g]primary portal vein hypoplasia, [h]castrated male, [i]present, [j]male, [k]female, [l]spayed female.
